# Supplementary material for: Predicting visual function by interpreting a neuronal wiring diagram
Source: Nature. 2024 Oct 2;634(8032):113–23. doi: 10.1038/s41586-024-07953-5 (PMC11446822; doi:10.1038/s41586-024-07953-5)
Supplement: Supplementary file 6 — CRF and ERF predictions for individual TmY4 and TmY9 cells. Analogous to Supplementary Data 3, but for TmY target types. Shown are the top four monosynaptic pathways, the strongest pathway passing through each of the top ten intermediary types (ranking from Extended Data Fig. 7), and the trisynaptic pathway Tm1–TmY–Dm3–TmY (see the section entitled Prediction of spatial normalization). [file 41586_2024_7953_MOESM6_ESM.zip › DataS4/TmY9qΓèÑ/720575940629429118.pdf]

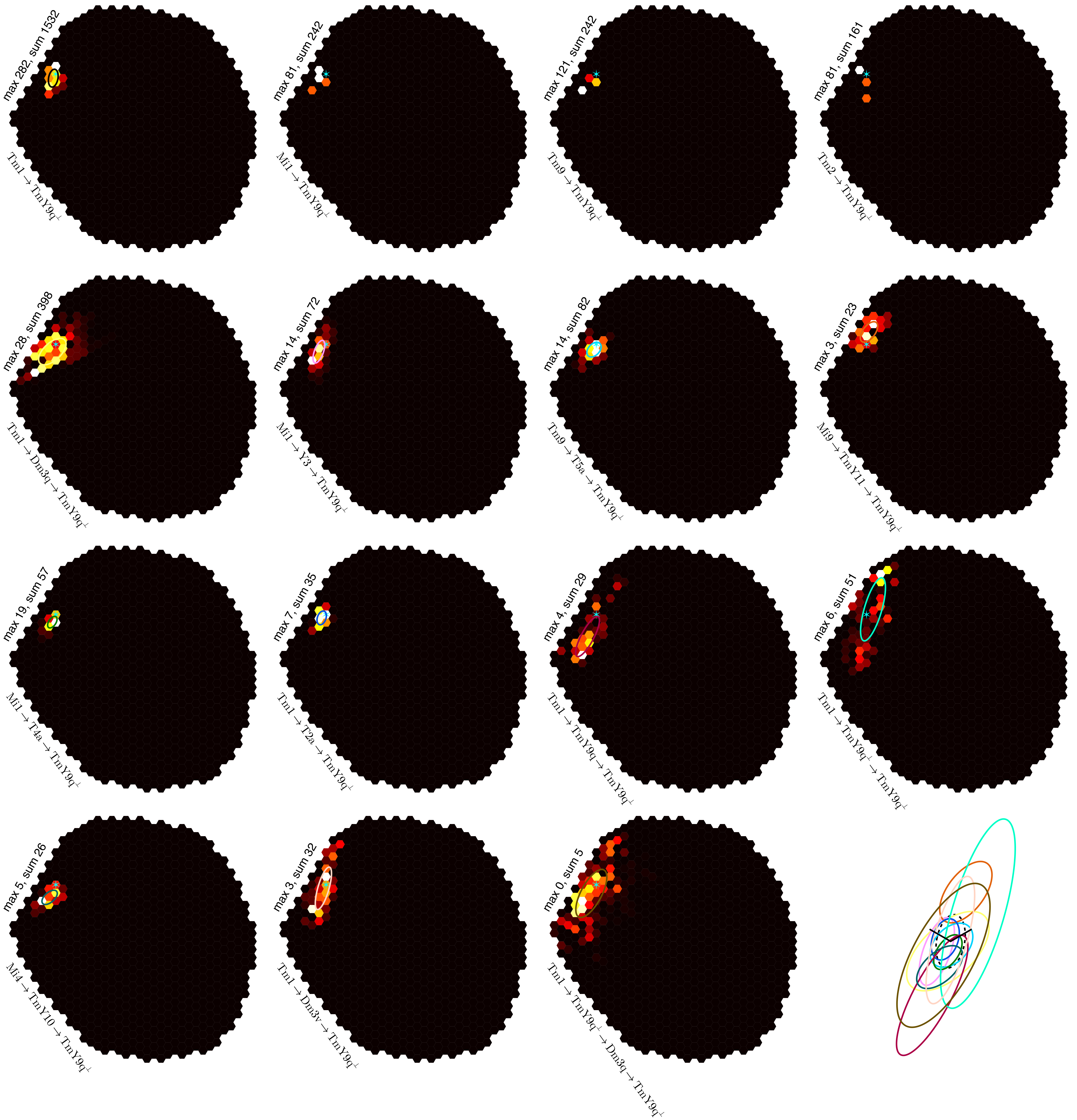
max 282, sum 1532
Tm1 → TmY9q⊥
max 81, sum 242
Mi1 → TmY9q⊥
max 121, sum 242
Tm9 → TmY9q⊥
max 81, sum 161
Tm2 → TmY9q⊥
max 28, sum 398
Tm1 → Dm3q → TmY9q⊥
max 14, sum 72
Mi1 → Y3 → TmY9q⊥
max 14, sum 82
Tm9 → T5a → TmY9q⊥
max 3, sum 23
Mi9 → TmY11 → TmY9q⊥
max 19, sum 57
Mi1 → T4a → TmY9q⊥
max 7, sum 35
Tm1 → T2a → TmY9q⊥
max 4, sum 29
Tm1 → TmY9q → TmY9q⊥
max 6, sum 51
Tm1 → TmY9q⊥ → TmY9q⊥
max 5, sum 26
Mi4 → TmY10 → TmY9q⊥
max 3, sum 32
Tm1 → Dm3v → TmY9q⊥
max 0, sum 5
Tm1 → TmY9q⊥ → Dm3q → TmY9q⊥
